# Case Report: Inhaled salbutamol in the successful treatment of life-threatening acute hyperkalaemia in an anaesthetised horse

**DOI:** 10.3389/fvets.2025.1663681

**Published:** 2026-01-16

**Authors:** Madelyn Rollet, Jana Flyps, Ingrid Vernemmen, Gunther van Loon, Stijn Schauvliege

**Affiliations:** 1 Department of Anaesthesia, Southern Counties Veterinary Specialists, Hampshire, United Kingdom; 2 Department of Large Animal Surgery, Anaesthesia and Orthopaedics, Ghent University, Ghent, Belgium; 3 Department of Internal Medicine, Reproduction and Population Medicine, Ghent University, Equine Cardioteam Ghent, Ghent, Belgium

**Keywords:** anaesthesia, atrial standstill, beta 2-agonists, cardiology, equine

## Abstract

Hyperkalaemia is an uncommon complication of general anaesthesia in healthy horses. This case report describes the occurrence of life-threatening acute hyperkalaemia in a 13-year-old, female French Trotter anaesthetised for experimental right and left atrial 3D electro-anatomical mapping. Intra-operative development of hyperkalaemia (7.55 mmol/L) (Ref. 3.00–4.00 mmol/L) with atrial standstill on ECG necessitated transvenous ventricular pacing while initial treatment with insulin and glucose was initiated. Plasma potassium levels continued to increase (8.00 mmol/L) prompting adjunctive treatment with 5 μg/kg of inhaled salbutamol and intravenous furosemide 0.93 mg/kg. Eight minutes after salbutamol administration, return of spontaneous atrial contraction was observed on echocardiography and plasma potassium concentration rapidly decreased on serial blood samples. To the authors’ knowledge, this is the first case report documenting the use of inhaled salbutamol in the treatment of life-threatening acute hyperkalaemia in an anaesthetised horse.

## Introduction

As the predominant intracellular cation, potassium is responsible for maintenance of normal resting cell membrane potential (RMP) (1). The body employs a range of homeostatic mechanisms to maintain potassium concentrations ([K+]) within a tight range. In the presence of certain pathologies, these homeostatic mechanisms may become overwhelmed, and elevated plasma [K+] (hyperkalaemia) can have important consequences.

Overall, the effects of hyperkalaemia on the heart are complex, and can broadly be divided into effects on the myocardium and on the conduction system. As [K+] increases, myocardial depolarisation becomes more difficult, eventually resulting in atrial standstill (AS). During AS, the atria are electrically silent and P waves disappear, although sinus node activity may persist (2). Conduction over the internodal tracts may initially be preserved, resulting in ventricular complexes of supraventricular origin (sinoventricular rhythm). However, as hyperkalaemia worsens, conduction across the atrioventricular node may also become impaired, resulting in atrioventricular block and bradycardia. Studies on hyperkalaemia show varying cardiovascular effects and are difficult to reproduce, likely reflecting complex ionic interactions and dynamic intra- and extra-cellular concentrations. Due to the effect of hyperkalaemia on the RMP, the severity of clinical signs increases in correlation with the elevation of plasma [K+] (3, 4). Initially, as [K+] begins to increase, the RMP becomes less negative, resulting in an increase in cell excitability (1). With continued [K+] elevation, the RMP will rise towards the threshold membrane potential, triggering an action potential. However, subsequent repolarisation may be impaired (1). Severe hyperkalaemia is therefore life-threatening due to its impact on cardiac cells, resulting in bradycardia, arrhythmias, and ultimately asystole. Characteristic ECG changes occur at certain ranges of increased [K+] (5). Tenting of T waves and shortening of the QT-interval may be observed with mild hyperkalaemia, due to abnormally rapid repolarisation of cardiac cells (1). As [K+] further increases, atrial conduction ceases, characterised by absence of P waves and sinoventricular bradycardia (1). In extremis, the QRS and T waves merge to create a sine wave, eventually progressing to ventricular fibrillation or asystole. However, ECG changes observed do not always correlate with plasma [K+] (6).

Hyperkalaemia during general anaesthesia (GA) of veterinary patients including cats (7), dogs (8) and horses (9) is increasingly reported, with several possible causes. These can be grouped into: increased potassium intake, decreased urinary excretion, translocation of potassium from the intracellular fluid (ICF) to the extracellular fluid (ECF) and pseudo-hyperkalaemia. Increased intake may occur iatrogenically following potassium supplementation. Reduced urinary excretion often occurs with urinary obstruction or bladder rupture. Translocation of potassium from the ICF to the ECF can result from pathophysiological factors or certain drugs. Equine Hyperkalaemic Periodic Paralysis (HYPP) is a genetic mutation in Quarter horses affecting skeletal muscle sodium channels, causing translocation of potassium from ICF to ECF, leading to hyperkalaemia (10). Various drugs, like alpha-2 adrenergic agonists are implicated in the development of hyperkalaemia (11).

Treatment of hyperkalaemia is multimodal and depends on the severity of the [K+] increase and the extent of the cardiac impact. The mainstay of treatment focuses on identifying and addressing the underlying cause. Potassium intake and hyperkalaemia-promoting drugs, such as potassium sparing diuretics, should be discontinued.

Intravenous (IV) calcium gluconate stabilises the membrane potential by raising the threshold membrane potential, restoring the gradient between the resting and threshold membrane potential (12). This attenuates the clinical signs and allows time to address the hyperkalaemia.

Intravenous fluid therapy (IVFT) should be initiated using crystalloids with low [K+] or potassium-free solutions (e.g., 0.9% saline). While 0.9% saline is potassium-free, caution is warranted due to the potential for hyperchloraemic acidosis, which may exacerbate hyperkalaemia.

In patients under GA, respiratory acidosis due to hypoventilation can exacerbate hyperkalaemia (13). Maintaining end-tidal carbon dioxide (ETCO_2_) levels at the low end of the reference interval through hyperventilation may be beneficial. Metabolic acidosis may also potentiate hyperkalaemia, and treatment with sodium bicarbonate should be considered (14).

Insulin reduces [K+] via translocation of potassium from the ECF into the ICF (1). To mitigate the risk of hypoglycaemia, insulin is typically administered alongside glucose IV, unless hyperglycaemia is present. While insulin and glucose therapy is commonly used in veterinary practice, some clinicians may prefer initial glucose administration alone due to its independent potassium-lowering effect and lower risk profile. Insulin and glucose combinations typically reduce [K+] within 30–60 min (1).

Loop diuretics such as furosemide may increase potassium elimination. Though commonly used in the management of acute hyperkalaemia in humans (15), no studies to date have assessed their efficacy for hyperkalaemic veterinary patients. Other treatments including potassium binding agents (resin polystyrene sulfonate) and dialysis have been reported but are less readily available (1).

Beta-2 agonists, such as salbutamol, stimulate the sodium-potassium-ATPase pump, promoting cellular potassium uptake (16). While commonly used for acute hyperkalaemia in human medicine alongside glucose and insulin, they are rarely considered in veterinary emergencies, possibly due to lack of knowledge, administration challenges, or concerns about hypotensive effects (17), particularly with IV use.

## Case presentation

A 13-year-old 540 kg female French Trotter was anaesthetised for a study on 3D electro-anatomical cardiac mapping via transvenous catheter placement and transseptal puncture, approved by the ethical committee. Pre-anaesthetic clinical examination revealed no abnormalities.

Heparin (Heparin, LEO) 80 IU/kg, IV was administered 50 min prior to GA induction. Premedication included romifidine (Rominervin^®^, Dechra) 80 μg/kg, IV and morphine (Morphine HCl, Sterop) 0.1 mg/kg, IV. A 12 G catheter was inserted into the left jugular vein and flunixin meglumine (Emdofluxin, Emdoka) 1.1 mg/kg, IV given. Anaesthesia was induced with ketamine (Ketamidor^®^, Ecuphar) 2.2 mg/kg, IV and midazolam (Dormazolam^®^, Dechra) 0.06 mg/kg, IV. The patient was placed in left lateral recumbency. Anaesthesia was maintained with isoflurane (Isoflutek^®^, Karizoo) (1.0–1.3% end tidal) in oxygen (1.5–2 L/min) delivered via a circular rebreathing circuit/large animal ventilator (Tafonius, Vetronic Services LTD) using assisted controlled ventilation (tidal volume: 7 L, peak inspiratory pressure: 28–36 mmHg, positive end expiratory pressure: 5 cmH_2_O, respiratory rate: 7 brpm). Ringer’s Lactate (Vetivex^®^ Veterinary Lactated Ringer’s, Dechra) was infused IV. Monitoring included pulse-oximetry, ECG, invasive arterial blood pressure (IBP), capnography, fraction inhaled/exhaled gasses/vapours, and temperature. Procaine benzylpenicillin (PENI-Kel, Kela) 21,000 IU/kg, intramuscular (IM) was administered. A urinary catheter remained in-situ throughout GA. Systemic hypertension (mean arterial pressure: 130 mmHg) was noted 10 min post-induction, and acepromazine (Tranquinervin^®^, Dechra) 0.01 mg/kg, IM was given.

**Figure 1:**
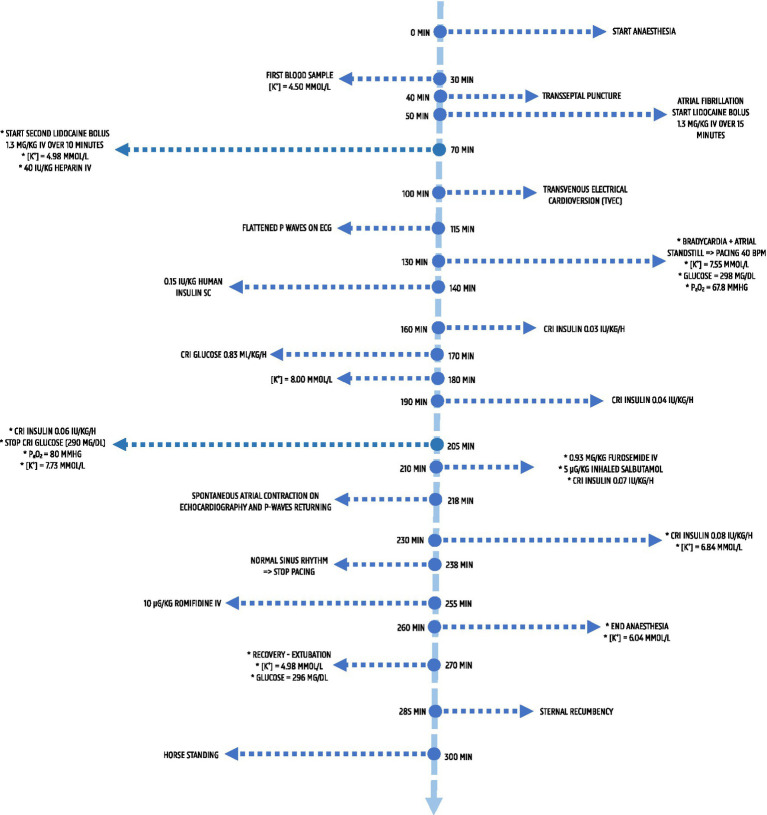
Timeline of key clinical events, interventions and blood gas results during anaesthesia. The timeline spans induction through recovery, showing blood gas and electrolyte values (potassium, glucose, pO_2_), onset of atrial fibrillation, transvenous electrical cardioversion (TVEC), progression to atrial standstill, initiation of treatment for hyperkalaemia and restoration of sinus rhythm prior to recovery.

Arterial blood-gas samples (ABG) were primarily analysed using the ABL-9 blood gas analyser (Radiometer). Two samples were assessed with the RAPIDpoint^®^ 500^e^-system (Siemens Healthineers), which also measured blood glucose and lactate. Additional glucose analysis was conducted with the ALPHATrak^®^ 2 glucometer (Zoetis). Table 1 summarises the results, and Figure 1 outlines the timescale of events.

**Table 1:** Sequential arterial and venous blood gas analyses obtained from the horse during anaesthesia and recovery.

Sample number	1	2	3	4	5	6	7	8	9	10	11	12	13
Anaesthesia time (minutes post-induction)	30	70	130	140	155	180	190	205	220	230	245	260 Immediately prior to recovery	10 Post-anaesthesia
Sample type	Arterial	Arterial	Arterial	Arterial	Arterial	Arterial	Arterial	Arterial	Arterial	Arterial	Venous	Venous	Venous
Analyser	Radiometer	Radiometer	RAPIDpoint 500e	Radiometer	Radiometer	Radiometer	RAPIDpoint 500e	Radiometer	Radiometer	Radiometer	Radiometer	Radiometer	RAPIDpoint 500e
pH	7.40	7.41	7.50	7.37	7.39	7.43	7.45	7.44	7.45	7.46	7.32	7.44	
pCO_2_ (mmHg)	49.8	49.8	43.4	56.7	53.7	49.1	41.0	48.6	47.2	46.1	67.9	49.3	
PO_2_ (mmHg)	301	105	67	61	64	69	–	80	151	166	39	56	
SO_2_ (%)	99.8	97.8	–	90.0	91.4	93.7	–	95.8	99.2	99.3	60.5	89.3	
HCO_3_ (mmol/L)	31.1	31.7	33.1	32.5	32.5	32.4	28.3	32.6	32.7	32.4	35.2	33.2	
tCO_2_ (mmol/L)	32.6	33.2	–	34.2	34.1	33.9	–	34.1	34.2	33.8	37.3	34.7	
Base excess	5.9	6.6	9.0	6.5	6.9	7.4	4.1	7.7	8.0	7.8	8.4	8.1	
HCT (%)	27	24	29	33	32	30	33	29	28	30	29	33	
Na + (mmol/l)	136	135	128	132	131	131	129	131	131	132	133	134	
K + (mmol/L)	4.50	4.98	7.55	7.29	7.67	8.00	7.55	7.73	7.26	6.84	6.76	6.04	4.98
Cl- (mmol/L)	100	99	96	95	96	96	96	96	96	96	95	97	
iCa^2+^ (mmol/L)	1.48	1.41	1.33	1.32	1.29	1.28	1.25	1.26	1.23	1.20	1.25	1.17	
Glucose (mg/dL)			298	270 (AlphaTRAK2)		195 (AlphaTRAK2)	290			326 (AlphaTRAK 2)			296
Lactate (mmol/L)			1.33				1.74						

Values are shown at specified time points post induction of general anaesthesia and immediately post-anaesthesia. Parameters include pH, pCO_2_, pO_2_, oxygen saturation (SO_2_), bicarbonate (HCO_3_^−^), total CO_2_ (tCO_2_), base excess, haematocrit (HCT), electrolytes (Na^+^, K^+^, Cl^−^, ionised Ca^2+^), glucose and lactate. Note the progressive hyperkalaemia (K^+^ rising to 8.00 mmol/L).

**Figure 2:**
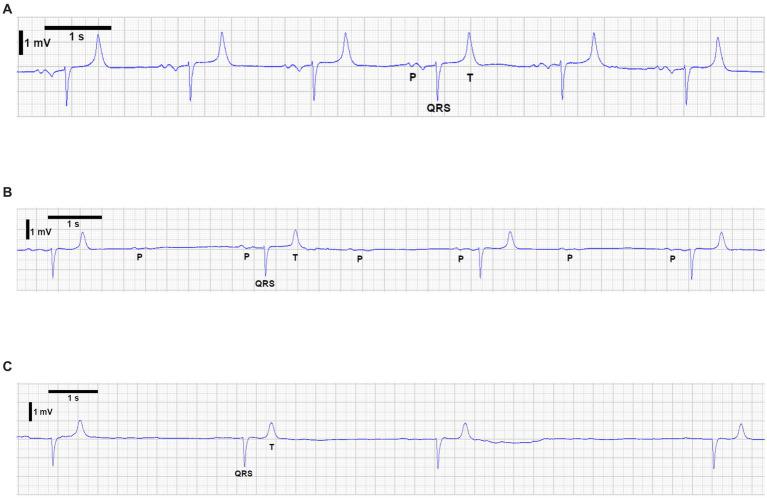
Electrocardiographic (ECG) changes observed in the horse during anaesthesia in association with hyperkalaemia. **(A)** Sinus rhythm after successful TVEC. **(B)** Flattening of P waves, with intermittent non-conducted P waves and bradycardia. **(C)** Atrial standstill consistent with severe hyperkalaemia.

Considering induction of anaesthesia as T0, an initial ABG collected at T30 showed no abnormalities except [K+] of 4.50 mmol/L. Atrial fibrillation developed at T50 due to the intracardiac electrophysiological study. Attempts to restore sinus rhythm with two boluses of lidocaine (Lidor^®^, Richter Pharma) 1.3 mg/kg over 15 min, IV, administered at T50 and T70, failed, but IBP remained stable. At T70, plasma [K+] was 4.98 mmol/L. In the absence of ECG changes suggestive of hyperkalaemia, treatment was deferred, and subsequent ABG monitoring continued. Heparin 40 IU/kg, IV was given at T70 to maintain activated clotting time above 300 s. At T100, transvenous electrical cardioversion (TVEC) successfully restored sinus rhythm (Figure 2A).

Subsequently, ECG changes (flattened P waves, second-degree atrioventricular block, bradycardia) were noted at T115 (Figure 2B). At T130, profound bradycardia (9 bpm) with AS (Figure 2C) occurred. Ventricular pacing at 40 bpm was initiated, and the study was terminated. ABG revealed hyperkalaemia (7.55 mmol/L), hyperglycaemia (298 mg/dL), and hypoxaemia (PaO_2_ 67.80 mmHg). To treat hypoxaemia, the tidal volume was increased to 8.5 L. Ringer’s Lactate (Vetivex^®^ Veterinary Lactated Ringer’s, Dechra) was replaced with 0.9% saline (0.9% sodium chloride, Baxter). Insulin (Humulin^®^ R, Lilly) 0.15 IU/kg, subcutaneously (SC) was given at T140, and a constant rate infusion (CRI) of insulin (Humulin^®^ R, Lilly) 0.03 IU/kg/h was started at T160 due to persistent hyperkalaemia (7.67 mmol/L at T155). ABG was then performed every 10–15 min to monitor [K+] and blood glucose.

Potential causes of hyperkalaemia were assessed. Urethral obstruction, bladder rupture, and iatrogenic potassium administration were ruled out. The urinary catheter was patent, with normal urine output. Abdominal ultrasound confirmed urinary bladder integrity and absence of free fluid.

At T170 a glucose CRI (Glucose 30%, Kela) 0.83 mL/kg/h was started. Blood gas analysis at T180 showed a blood glucose of 195 mg/dL. The insulin rate was increased to 0.04 IU/kg/h at T190.

A blood sample at T205 showed an improvement in PaO_2_ to 80 mmHg and a glucose concentration of 290 mg/dL. The glucose CRI was discontinued. However, because [K+] had only decreased to 7.73 mmol/L the insulin CRI was increased to 0.06 IU/kg/h. At T210, inhaled salbutamol (Ventolin, GSK) 5 μg/kg, over 12 breaths, via the Y-piece of the breathing system and furosemide (Dimazon^®^, MSD Animal Health) 0.93 mg/kg, IV were administered. The insulin CRI was increased to 0.07 IU/kg/h. Eight minutes later, spontaneous atrial depolarisation and contraction were beginning to return (ECG/echocardiography) with occasional P waves observed. At T238, sinus rhythm returned 28 min post-salbutamol/furosemide (90 min post-onset of AS), and ventricular pacing was discontinued. The [K+] had dropped to 7.26 mmol/L and PaO_2_ increased to 151 mmHg at T220. At T230, [K+] was 6.84 mmol/L and blood glucose 326 mg/dL. The insulin CRI was increased to 0.08 IU/kg/h. Anaesthesia was maintained for another 30 min to confirm that heart rate and rhythm remained stable. A final venous blood sample before recovery at T260 documented [K+] at 6.04 mmol/L.

Romifidine (Rominervin^®^, Dechra) 10 μg/kg, IV was administered. No adverse cardiac effects were observed, so the horse was hoisted into the recovery box and positioned in left lateral recumbency. IVFT with 0.9% saline and insulin CRI 0.08 IU/kg/h were continued. Phenylephrine 1% solution (Minims Phenylephrine hydrochloride, Bausch and Lomb) 5 mL per nostril was given to reduce intra-nasal oedema. Oxygen 15 L/min was delivered via the endotracheal tube until extubation (10 min post-GA) and intranasally thereafter. Recovery was assisted with head and tail ropes. At extubation, a venous blood sample showed [K+] at 4.89 mmol/L and glucose at 296 mg/dL.

The horse moved into sternal recumbency 25 min post-GA and after several attempts, stood successfully 40 min post-GA. Recovery was scored based on a scoring system produced by Schauvliege et al. (18), and was scored as moderate (3/6) with weakness and ataxia. Post-anaesthetic monitoring of [K+] was continued. IVFT (0.9% saline) and the insulin CRI 0.08 IU/kg/h were maintained. Venous blood samples at 30, 90, and 270 min post-recovery showed [K+] of 3.74, 3.52, and 2.68 mmol/L and glucose of 200, 185, and 70 mg/dL, respectively. IVFT 0.9% saline and insulin CRI were discontinued upon normokalaemia. Post-operatively, the horse received procaine benzylpenicillin (PENI-Kel, Kela) 21,000 IU/kg, IM for 2 days, flunixin meglumine (Emdofluxin, Emdoka) 1.1 mg/kg orally for 1 day, and enoxaparin (Clexane®, Sanofi) 0.28 mg/kg, SC for 7 days. No post-operative complications were observed.

## Discussion

The cause of hyperkalaemia was unclear. Without pre-anaesthetic blood sampling, it is unknown if mild hyperkalaemia existed prior. The rapid rise from 4.50 mmol/L at initial ABG to severe hyperkalaemia suggests an intra-operative cause. Pseudo-hyperkalaemia was excluded due to the presence of ECG changes compatible with hyperkalaemia. No evidence of increased potassium intake or reduced excretion was found. HYPP is unlikely, as affected horses trace to the Quarter Horse sire “Impressive” (19). However, genetic testing is needed to fully rule out this diagnosis.

Procedural factors were also considered as potential contributors to the development of hyperkalaemia. The transseptal puncture was performed at T40 and involves minimal, localised tissue trauma; therefore a delayed [K+] release as a result of this step was considered unlikely. TVEC was performed at T100, approximately 30 min before the onset of bradycardia and atrial standstill. Although TVEC delivers brief, high-energy shocks that could theoretically cause transient myocardial cell membrane disruption and potassium efflux, this mechanism has not been reported in either human or equine patients. Baseline cardiac troponin I (cTnI) was negative, with only mild increases within the reference range (0–0.06 ng/mL) observed following the procedure: 0.02 ng/mL after transseptal puncture, 0.019 ng/mL 4 h post-transseptal puncture, and 0.045 ng/mL 8 h post-transseptal puncture (Medilab). Reported concentrations of cTnI >0.03 ng/mL are observed more frequently in horses with myocardial disease (20). These findings suggest only minimal myocardial irritation, consistent with the limited tissue trauma expected from transseptal puncture and TVEC, and make significant myocardial damage an unlikely source of the hyperkalaemia.

Translocation of potassium from the ICF to the ECF was the likely cause of hyperkalaemia. In humans an increase in [K+] by ~ 0.39 mmol/L is reported for every 7.5 mmHg increase in PaCO_2_ (13). While respiratory acidosis may increase plasma [K+] via potassium efflux, ventilation was assisted and PaCO_2_ remained below 60 mmHg, making hypercapnia an unlikely cause. Literature on respiratory acidosis and hyperkalaemia remains conflicting in both humans and dogs (21–25), with no clear consensus on its effect on plasma [K+].

It seems most likely that the hyperkalaemia was due to one or more of the drugs administered peri-anaesthesia. In humans, various drugs can cause hyperkalaemia by promoting potassium shifts or reducing renal excretion (26). NSAIDs can induce hyperkalaemia via renin-aldosterone-angiotensin system inhibition in humans (26), though veterinary reports are lacking. Pre-anaesthetic flunixin meglumine is unlikely implicated here, as severe hyperkalaemia and ECG changes appeared 130 min post-induction.

Alpha-2 agonists may cause hyperkalaemia via hyperglycaemia and hypoinsulinaemia (11). Romifidine pre-medication may have been associated with the development of hyperkalaemia. Ketamine combined with medetomidine may cause hyperkalaemia by disrupting alpha/beta adrenoreceptor balance, potentially inducing autonomic insufficiency (27). A similar association with romifidine is plausible but unconfirmed. No literature links opioids or midazolam to hyperkalaemia.

Isoflurane is linked to hyperkalaemia in humans (28) and may promote potassium efflux (29), making it a possible factor in this case. No evidence exists on whether IV lidocaine promotes hyperkalaemia, though hyperkalaemia may exacerbate lidocaine’s cardiotoxic effects (30). In this case, hyperkalaemia had not been observed at the time of lidocaine administration.

Hyperkalaemia occurs in 7% of humans receiving heparin (31), linked to its reversible inhibition of aldosterone synthesis and angiotensin II receptors (26, 32). However, this complication typically arises several days post-treatment (33) while in the current case, atrial standstill occurred 60 min after heparin administration. Procaine benzylpenicillin, which contains potassium, may cause significant [K+] alterations at high doses in humans (34).

In this case, the ECG changes were compatible with life-threatening hyperkalaemia (7.55 mmol/L). Earlier ABG samples showed a gradual potassium increase without ECG changes. Intermediate ECG changes (flattened/non-conductive P waves, bradycardia) (Figure 2B) briefly preceded profound bradycardia (9 bpm) and AS (Figure 2C).

Initial treatment included 0.9% saline, insulin and glucose. Ringer’s Lactate was discontinued in favour of potassium-free 0.9% saline. While 0.9% saline can cause hyperchloraemic acidosis, neither metabolic acidosis nor hyperchloraemia were observed. Despite 60 min of insulin and glucose infusions, serum potassium levels remained high, with no effect on AS.

To the authors’ knowledge this is the first case report documenting use of inhaled salbutamol in treatment of life-threatening hyperkalaemia in an anaesthetised horse. No recommended dosage of salbutamol exists for the treatment of hyperkalaemia in equids. An aerosolised dose of 2 μg/kg is reportedly effective to treat hypoxaemia in horses under GA (35). Via the Y piece of the breathing system, 5 μg/kg was administered on inhalation over 12 breaths. Eight minutes later, atrial contraction was observed on echocardiography and within 28 min, sinus rhythm had resumed allowing cessation of ventricular cardiac pacing. Within 15 min [K+] reduced by 0.47 mmol/L and continued to decline further until recovery from GA.

Beta-2 agonists are used to treat hyperkalaemia in people. They stimulate sodium-potassium-ATPase activity, facilitating intracellular potassium influx (16). In children, use of IV doses as low as 4 μg/kg reduce [K+] (36). In adult humans, the dosage of salbutamol for treatment of hyperkalaemia ranges from 0.5 mg IV to 10 mg nebulised. Onset of action is within 30-min, with peak effect occurring at approximately 30 and 90 min post-IV and nebulised dose, respectively (37). Salbutamol has been shown to produce a reduction in [K+] of 0.87–1.40 mmol/L post-IV administration (37–39) and 0.53–0.98 mmol/L post-nebulisation in people (37, 40, 41). Nebulised doses of 20 mg are more efficacious at 120-min than 10 mg (37, 40). No difference has been observed between insulin with glucose and either intravenous or nebulised salbutamol (37, 38, 42), nor in maximum effect between IV and nebulised salbutamol (37, 39, 43, 44). Approximately 12–40% of human patients are unresponsive to treatment with salbutamol (37, 42, 45), and it is recommended that salbutamol is always used in conjunction with insulin. Furthermore, human studies demonstrate that combination of salbutamol, insulin and glucose outperforms insulin alone (37, 38, 42).

Inhaled salbutamol reduced whole blood [K+] in a dose-dependent manner in 10 healthy dogs (46). Similarly, in an experimental study of hyperkalaemia in rabbits, norepinephrine and salbutamol decreased serum [K+] (47).

Aerosolised salbutamol (2 μg/kg) also reduces arterial [K+] in healthy anaesthetised horses (48). However, the duration of this effect has not been assessed (48). Adverse effects of beta-2 agonists include tachycardia, arrhythmias, and tremors in humans. Hypotension is also reported in anaesthetised horses (17). Further research is needed in veterinary species, including equids, to evaluate salbutamol for acute hyperkalaemia treatment.

A dose of furosemide, 0.93 mg/kg, IV was given around the time of inhaled salbutamol administration. Furosemide, a loop diuretic, inhibits the sodium-potassium-chloride co-transporter in the ascending loop of Henle reducing their reabsorption, and increasing potassium excretion in urine (49).

In humans, furosemide’s potassium-reducing action has a delayed response, with an onset of action in 5–30 min and a duration of 2–6 h (50). No studies have yet evaluated loop diuretics’ effectiveness in excreting potassium in hyperkalaemic animals. Although noted as a potential treatment for hyperkalaemia in animals (51), no reports document furosemide’s use for acute cases in veterinary patients.

The cause of insufficient [K+] decline after insulin and glucose administration in this case is unclear. Given insulin’s dose-dependent effects on potassium translocation, the inadequate response could be attributed to insufficient dose. However, judicious insulin rates are recommended due to the risk of resultant hypoglycaemia. Another potential cause of this lack of response could be insulin-resistance in insulin-targeting tissues. The association between insulin dysregulation and equine metabolic syndrome is well-recognised (52) and the prevalence of hyperinsulinaemia has been reported as 18% in healthy, non-laminitic horses in one study (53). In people, serum [K+] is often increased in poorly controlled type-2 diabetes mellitus with insulin-resistance (54). The presence of basal hyperinsulinaemia was not established, but no other clinical signs of equine metabolic syndrome were observed.

## Conclusion

This case report highlights the rapidity with which severe, acute hyperkalaemia can occur in horses, with life-threatening clinical signs, necessitating prompt treatment to normalise [K+]. The exact cause in this anaesthetised horse remains unclear but is likely multifactorial, possibly linked to drug administration. When an inciting cause is identified, treatment should focus on addressing it. While insulin and glucose remain the mainstay of treatment for hyperkalaemia in veterinary patients, multimodal adjuncts like salbutamol or furosemide may be useful, especially in refractory cases. Further research is needed on their efficacy in equids and other veterinary species.

## Data Availability

The original contributions presented in the study are included in the article/supplementary material, further inquiries can be directed to the corresponding author.
